# Screening of Vietnamese medicinal plants for NF-κB signaling inhibitors: Assessing the activity of flavonoids from the stem bark of *Oroxylum indicum*

**DOI:** 10.1016/j.jep.2014.10.012

**Published:** 2015-01-15

**Authors:** Thi Van Anh Tran, Clemens Malainer, Stefan Schwaiger, Tran Hung, Atanas G. Atanasov, Elke H. Heiss, Verena M. Dirsch, Hermann Stuppner

**Affiliations:** a Institute of Pharmacy/Pharmacognosy, Center for Molecular Biosciences Innsbruck, University of Innsbruck, Innrain 80/82, Innsbruck 6020, Austria; b Department of Pharmacognosy, University of Vienna, Althanstrasse 14, Vienna 1090, Austria; c Department of Pharmacognosy, Faculty of Pharmacy, University of Medicine and Pharmacy of Ho Chi Minh City, 41 DinhTienHoang Street, Ho Chi Minh City, Vietnam

**Keywords:** NF-κB, *Oroxylum indicum*, Hispidulin, Baicalein, Chrysin, Oroxylin A

## Abstract

**Ethnopharmacological relevance:**

Seventeen plants used in Vietnamese traditional medicine for the treatment of inflammatory disorders were screened for NF-κB inhibitory activity. *Oroxylum indicum*, which exhibited activity, was investigated in detail.

**Materials and methods:**

Forty plant extracts from 17 species were prepared by maceration using dichloromethane and methanol and were tested (10 µg/mL) to evaluate their ability to inhibit NF-κB activation using TNF-α-stimulated HEK-293 cells stably transfected with a NF-κB-driven luciferase reporter. The active extract of *Oroxylum indicum* was subsequently fractionated by different chromatographic techniques. After isolation, all single compounds were identified by spectroscopic methods and assessed for NF-κB inhibitory effects.

**Results:**

The dichloromethane extracts obtained from *Chromolaena odorata* leaves and the stem bark of *Oroxylum indicum* showed distinct inhibitory effects on NF-κB activation at a concentration of 10 µg/mL. The active extract of *Oroxylum indicum* was subjected to further phytochemical studies resulting in identification of four flavonoid aglyca and six flavonoid glycosides. Pharmacological evaluation of the obtained compounds identified oroxylin A as the most active substance (IC_50_=3.9 µM, 95% CI: 3.5–4.4 µM), while chrysin and hispidulin showed lower activity with IC_50_=7.2 µM (95% CI: 6.0–8.8 µM) and 9.0 µM (95% CI: 7.9–10.2 µM), respectively. Interestingly, in this study the activity of baicalein (IC_50_=28.1 µM, 95% CI: 24.6–32.0 µM) was weak. The isolated glycosides showed no inhibitory activity when tested at a concentration of 30 µM. Quantification of the four active flavonoids in extracts and plant materials suggested that oroxylin A contributes to the NF-κB inhibitory activity of the stem barks of *Oroxylum indicum* to a greater extent than baicalein which was thought to be responsible for the anti-inflammatory activity of this plant.

**Conclusions:**

The screening presented in this study identified the dichloromethane extracts of *Chromolaena odorata* and *Oroxylum indicum* as promising sources for NF-κB inhibitors. Hispidulin, baicalein, chrysin and oroxylin A, isolated from *Oroxylum indicum*, were identified as inhibitors of NF- κB activation.

## Graphical abstract

**Figure:**
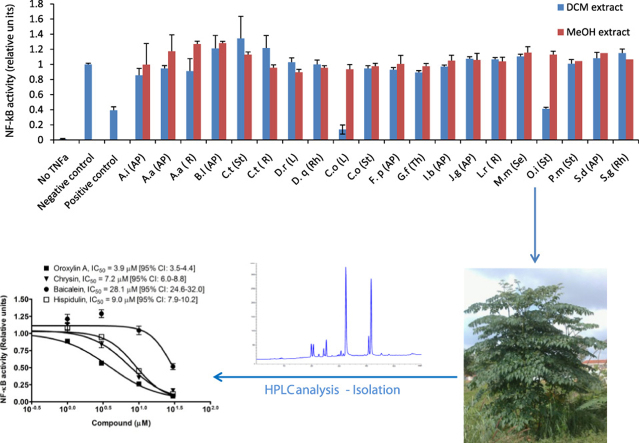

## 1 Introduction

Inflammation is a response of organisms to the presence of pathogens and chemical or mechanical injury. Although it is a defense mechanism, the complex events and mediators in the uncontrolled inflammation reaction can induce or sustain a pathologic process involved in different diseases *e.g*. arthritis, atherosclerosis, allergy, the metabolic syndrome, sepsis and auto-immune diseases as well as cancer (Libby et al., 2002; Allavena et al., 2008). Initiation and promotion of inflammatory processes require synthesis of effector proteins such as cell adhesion molecules (VCAM-1, ICAM-1), inducible enzymes (COX-2 and iNOS), chemokines (MCP-1, MIP-1α), cytokines and growth factors (tumor necrosis factor alpha (TNF-α), interleukins) (Sacca et al., 1997; Eppihimer, 1998). Thus, one strategy to block inflammation is to inhibit inflammatory mediators on the transcriptional level. Among the involved transcription factors, nuclear factor kappa B (NF-κB) plays a prominent role. Therefore, the NF-κB signaling pathway represents an established target for anti-inflammatory drug discovery (Sun and Andersson, 2002; Gupta et al., 2010). Besides synthetic compounds, several natural products have been identified to inhibit NF-κB (Bremner and Heinrich, 2002, 2005; Calixto et al., 2003; Gupta et al., 2010; Baumgartner et al., 2011).

In Vietnam, traditional medicine has a long established history and plays an important role in the health care system. Vietnam׳s territory with an area of more than 3,30,000 square kilometers has a number of key ecosystems including a variety of marine and coastal habitats, wetland, and two important river deltas and is known as a hot spot of biodiversity covering more than 12,000 plant species (Nguyen et al., 2007). Many of these plants have been used to treat diseases and traditional knowledge is still preserved. In 2009, Nguyen and You screened 87 plants used in Vietnamese traditional medicine for inhibitory activity on NF-κB activation and identified seven methanolic extracts as potential inhibitors of this pathway. In a continuing effort to rationalize and assess the activity of medicinal plants in Vietnam for inflammatory diseases, we conducted a screening of 17 plants which are listed in the textbook “Medicinal plants and animals in Vietnam (National Institute of Medicinal Materials, 2004)”. Two of the plant extracts showed promising inhibitory activity on NF-κB activation: the dichloromethane extracts of *Oroxylum indicum* and *Chromolaena odorata*. In 2010, Siriwatanametanon et al. found that the ethyl acetate extract of *Oroxylum indicum* possesses NF-κB inhibitory activity; however, identification of the responsible compound(s) was not described.

## 2 Materials and methods

### 2.1 Plant material

The plant material used in this study was collected from different locations in the south of Vietnam between March and September 2010. The collected species were identified and authenticated by taxonomists from the Department of Pharmacognosy, Faculty of Pharmacy, University of Medicine and Pharmacy of HoChiMinh city. Voucher specimens of plants are deposited in the herbarium of the Pharmacognosy Department at University of Medicine and Pharmacy of HoChiMinh city (Table 1). Additional samples of the stem bark of *Oroxylum indicum* were collected in CuChi (sample Oro-1), in the botanical garden of Faculty of Pharmacy (HoChiMinh city; sample Oro-2), while sample Oro-3 and Oro-4 were collected in BinhPhuoc. All plant samples were air-dried and finally ground to a fine powder before further processing.

### 2.2 Extraction

#### 2.2.1 Preparation of extracts for screening

Finely ground plant material (5 g) was extracted with 50 mL dichloromethane by sonication for 10 min at room temperature. The plant material was recovered by filtration and the process was repeated three times with fresh solvent. The obtained solutions were combined and evaporated to dryness using a rotavapor to give dichloromethane extracts. The residual plant material was air-dried and subsequently extracted with methanol using the same procedure as described above to yield the corresponding methanol extracts. All dried extracts were dissolved in dimethyl sulfoxide (DMSO) prior to bioactivity evaluation.

#### 2.2.2 Extraction of the stem bark of *Oroxylum indicum* for quantification purposes

Dried stem barks of *Oroxylum indicum* were cut into pieces and ground to a fine powder. The plant material (0.5 g) was extracted five times with 40 mL of methanol by sonication (15 min each, at ambient temperature) and then centrifuged at 3300 rpm for 7 min. Extracts were combined, evaporated under reduced pressure and subsequently re-dissolved in methanol, quantitatively transferred to a volumetric flask and adjusted to the final volume (10 mL) with methanol. Prior to injection, all solutions were filtered through cotton wool. Each sample solution was assayed in triplicate.

### 2.3 NF-κB activity and cell viability

HEK293/NF-κB-luc cells (Panomics, RC0014), a HEK293 cell line stably transfected with NF-κB luciferase reporter, were used to determine NF-κB activity and cell viability as previously described (Vogl et al., 2013). Briefly, cells were maintained at 37 °C and 5% CO_2_ atmosphere in Dulbecco׳s modified Eagle׳s medium (DMEM; Lonza, Basel, Switzerland) with 100 U/mL benzylpenicillin 100 μg/mL streptomycin, 2 mM glutamine, and 10% fetal bovine serum (FBS). Before seeding in 96-well plates, cells were stained for 1 h in serum-free medium supplemented with 2 μM Cell Tracker Green CMFDA (Invitrogen), a fluorescent probe that is retained inside living cells and thus can be used to monitor cell membrane integrity and cell viability (Markasz et al., 2007; Stern et al., 2008; Johnson-Lyles et al., 2010; Vogl et al., 2013). Cells were then plated in 96-well plates (4×10^4^ cells/well) in phenol red-free and FBS-free DMEM overnight. The following morning, cells were pretreated with the investigated samples, positive control (parthenolide, 10 or 5 µM; IC_50_ value: 1.5 µM, 95% CI 1.3–1.8 µM) or solvent vehicle (0.1% DMSO in culture medium) for 30 min and stimulated with 2 ng/mL TNF-α for 4 h. Afterwards, the cells were lysed with a luciferase lysis buffer (Promega; E1531) and the luminescence of the firefly luciferase and the fluorescence of the Cell Tracker Green CMFDA were measured with a Genios Pro plate reader (Tecan, Grödig, Austria). For quantification of NF-κB activity, the luciferase-derived signal from the NF-κB reporter was normalized by the fluorescence signal derived from Cell Tracker Green CMFDA to account for differences in the cell number. Potential differences in viable cell numbers were detected by comparison of the Cell Tracker Green CMFDA fluorescence of the solvent vehicle treated cells and cells treated with the investigated samples.

### 2.4 Statistical analyses

Nonlinear regression (with sigmoidal dose response) was used to calculate the IC_50_ values using GraphPad Prism^®^ 4.03 (GraphPad Software, Inc.). Statistical differences were compared with ANOVA with Tukey׳s post-hoc test. *P* values<0.05 were considered to be significant.

### 2.5 Chromatography and NMR analysis

Fast Centrifugal Partition Chromatography (FCPC) (Kromaton, France) was equipped with Gilson 302/803 C pump system model 302 (Villiers-la-Bel, France).

LC for quantification—LC-parameter: HP 1090 system (Agilent, Waldbronn, Germany) equipped with autosampler, DAD and column thermostat; stationary phase: Zorbax SB-C18 column (150×4.6 mm, 3.5 µm particle size); mobile phase: solvent A: H_2_O with 0.5% formic acid (v/v); solvent B: methanol; DAD: 275 nm, temp.: 40.0 °C; injection volume: 5 μL; flow: 0.3 mL/min; composition during run: start: 40% B; 37 min: 70% B; 50 min: 75% B; 51 min: 98% B; 60 min: stop; post-time: 10 min.

LC–MS—LC-parameter: HP 1100 system (Agilent, Waldbronn, Germany) was equipped with auto-sampler, DAD and column thermostat. MS-parameters: Esquire 3000^plus^ (Bruker Daltonics, Bremen, Germany); ESI, temperature: 350 °C; dry gas: 10.00 L/min; nebulizer 40 psi; full scan mode: *m*/*z* 100–1500.

NMR: 1D- and 2D-experiments were measured on a Bruker DRX 300 (Bruker Biospin Rheinstetten, Germany) operating at 300.13 MHz (^1^H) and 75.47 MHz (^13^C) at 300 K (chemical shifts *δ* in ppm, coupling constants *J* in Hz); NMR solvent: MeOH-*d*_4_ or DMSO-*d*_6_ with 0.03% TMS (Eurisotop Gif-Sur-Yvette, France), which was used as an internal standard.

### 2.6 Extraction, purification and identification of pure compounds from *Oroxylum indicum*

Dried stem barks of *Oroxylum indicum* (Oro-1) (100 g) were ground to a fine powder and extracted with 500 mL methanol at room temperature (10 times). After removal of the solvent under reduced pressure, the resulting crude extract (16.10 g) was suspended in 300 mL of H_2_O and successively extracted with petroleum ether (300 mL×4), ethyl acetate (300 mL×4) and water saturated *n-*BuOH (300 mL×4). The resulting extracts were concentrated under reduced pressure, to give petroleum ether (0.72 g), ethyl acetate (0.85 g), *n-*butanol (2.74 g) and water (11.24 g) extracts. The ethyl acetate extract (0.85 g) was subjected to silica gel (40–63 μm, Merck, Darmstadt, Germany; 100 g) column chromatography, using a CH_2_Cl_2_/MeOH gradient with increasing amounts of MeOH as mobile phase. The eluate was monitored by TLC and combined to 19 fractions (O.E1 – O.E19). Fraction O.E3 (CH_2_Cl_2_/MeOH, 99.7: 0.3) was recrystallized in CH_2_Cl_2_ to yield compound **1** (37.62 mg). Fraction O.E7 (CH_2_Cl_2_/MeOH, 99.5: 0.5; 35.46 mg) was purified by Sephadex LH-20 column chromatography using MeOH as mobile phase, yielding 15.44 mg of compound **2**. Fraction O.E11 (CH_2_Cl_2_/MeOH, 97: 3; 91.24 mg) was rechromatographed by Sephadex LH-20 column chromatography using MeOH as mobile phase, yielding 8 fractions (OE.11.1 – OE.11.8) and compound **3** in fraction O.E11.5 (31.25 mg). Compound **4** (4.69 mg) was obtained by separation of fraction OE.11.7 by Sephadex LH-20 column chromatography with MeOH as mobile phase.

**Fig. 1:**
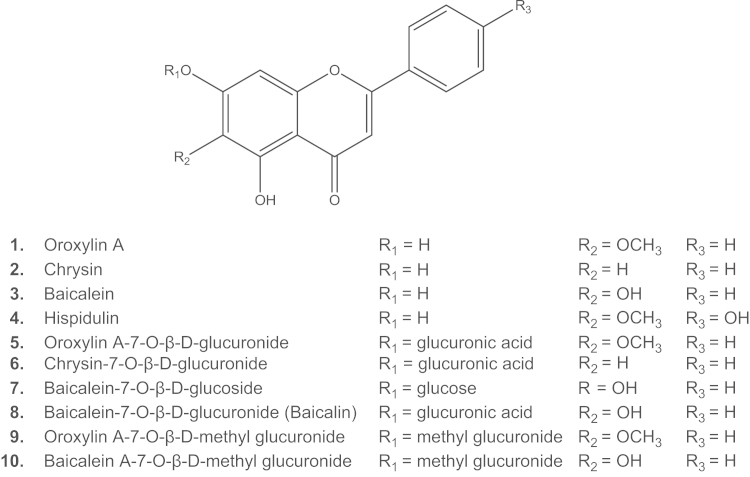
Structures of compounds (**1–10**) isolated from the stem bark of *Oroxylum indicum*.

Since the minor compounds of the ethyl acetate extract were also present in a higher amount in the *n-*butanol extract (verified by HPLC-MS-experiments), the isolation work was carried out using the *n-*butanol extract. The *n-*butanol extract was re-dissolved in methanol. The MeOH-soluble part was fractioned by Sephadex LH-20 column chromatography with MeOH as mobile phase to yield 10 fractions (OB.1–OB.10). Fraction OB.4 (338.60 mg) was purified by means of FCPC using a solvent mixture of chloroform, methanol, water (9.5:10:5, all v/v, lower phase:mobile phase) to yield compound **5** (26.68 mg) and compound **8** (13.59 mg). Subfraction OB.4.2 (28.70 mg) was further separated with Sephadex LH-20 column chromatography using a mixture of methanol and water (1+1, v/v) as mobile phase to obtain compound **10** (5.34 mg) and compound **7** (4.90 mg). The MeOH-insoluble part of the *n-*butanol extract was separated by FCPC using the solvent system ethyl acetate, ethylene glycol dimethyl ether and water (2:1:2, upper phase:mobile phase), yielding 11 fractions (OBI.1–OBI.11). Fraction OBI.2 (16.63 mg) and fraction OBI.5 (9.50 mg) were rechromatographed by Sephadex LH-20 column chromatography using a mixture of methanol and water (1:1, v/v) as mobile phase to afford compound **9** (8.91 mg) and compound **6** (3.55 mg), respectively (Fig. 1).

## 3 Results and discussions

### 3.1 NF-κB inhibitory activities of dichloromethane and methanol extracts of 17 Vietnamese medicinal plants

**Fig. 2:**
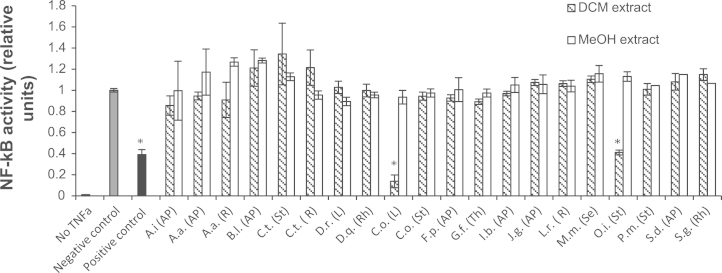
Effect of plant extracts (10 µg/mL) on NF-κB activation in HEK-293/NF-κB-luc cells; negative control: cells were treated with solvent vehicle (DMSO, 0.1%) and stimulated with TNF-α (2 ng/mL); positive control: cells were treated with parthenolide (10 µM) and stimulated with TNF-α. AP=aerial part; R=roots; St=stems; L=leaves; R=roots; Se=seeds; Th=thorns. The first letters of genus and species name of each plant were used to abbreviate plant names; see Table 1 for full names. Data points represent mean±S.E.M.; *n*=3, ^*^p<0.05 (ANOVA/Tukey) compared with negative control.

In a primary screen, 40 different extracts of 17 plants were analyzed for their potential to inhibit TNF-α induced NF-κB activation in HEK-293 cells at a concentration of 10 µg/mL. Results are presented in Fig. 2. Strongest inhibition of NF-κB activation was found for *Chromolaena odorata* (DCM extract of the leaves) and *Oroxylum indicum* (DCM extract of the stem bark). In contrast to a previous study (Nguyen and You, 2009) dichloromethane and methanol extracts of *Smilax glabra* at a concentration of 10 µg/mL did not show any effect, whereas the dichloromethane extract of *Chromolaena odorata* (at 10 µg/mL) was found to abrogate activation of NF-κB to a higher degree as the positive control (at 10 µM). In the study of Nguyen and You (2009) methanol extracts of both species exhibited some minor NF-κB inhibitory effects (<25% at 30 µg/mL). The rather strong effect of the dichloromethane extract of *Chromolaena odorata* in comparison to that of the methanol extract might be due to lipophilic constituents extractable by means of apolar solvents but not or only to some extent by polar ones. This is consistent with a recent investigation which identified six fatty acids as well as scutellarein tetramethyl ether from *Chromolaena odorata* (Tran et al., 2011) as inhibitors of NF-κB activation. Further differences in the obtained findings might be a result of discrepancies in preparation of extracts, the setup of the *in vitro* assay, the selected sample concentration, and varying contents of active substances in the plant raw material, depending on harvest time and other influences.

NFκB inhibitory effects of the ethyl acetate extract of *Oroxylum indicum* were already reported by Siriwatanametanon et al. (2010), but the active principles were not determined. In the present study the dichloromethane extract of *Oroxylum indicum* was investigated and identified also as an inhibitor of NF-κB activation. Thus, both dichloromethane and ethyl acetate extracts were subjected to further phytochemical analysis.

### 3.2 Isolation of single constituents from the active fraction of *Oroxylum indicum*

**Fig. 3:**
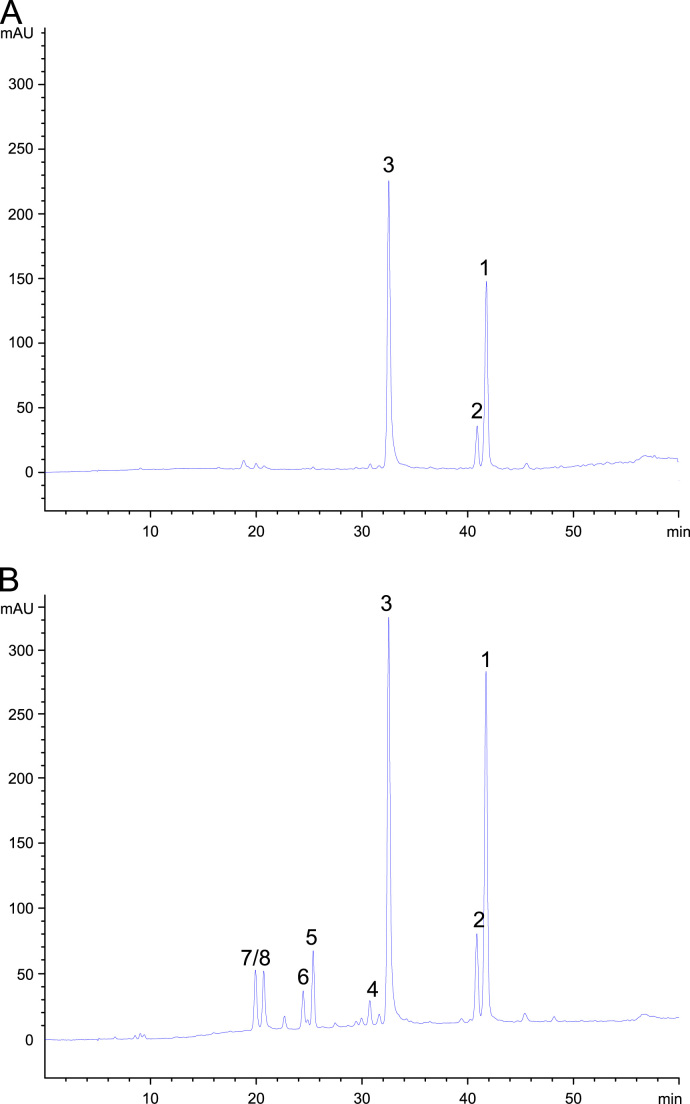
HPL chromatograms of (A) dichloromethane extract and (B) ethyl acetate extract monitored at wavelength of 275 nm; for HPLC conditions see Section 2. Compound assignment: 1—Oroxylin A; 2—Chrysin; 3—Baicalein; 4—Hispidulin; for other compounds see Fig. 1.

Fig. 3 shows HPLC-DAD chromatograms of dichloromethane and ethyl acetate extracts of the stem bark of *Oroxylum indicum*. Compounds **1**–**3** are the main constituents of both extracts, while compounds **4**–**8** were not present in the dichloromethane extract. Therefore, a large scale ethyl acetate extract was prepared. Since compounds **5**–**8** were found in higher amounts in the *n-*butanol extract, isolation of these compounds was conducted using the *n*-butanol extract. After several purification steps by means of silica gel column chromatography, gel-filtration chromatography and counter-current chromatography, four flavonoid aglyca (**1**–**4**) could be isolated from the ethyl acetate extract and six flavonoid glycosides from the *n-*butanol extract (**5**–**10**). ^1^H and ^13^C NMR, as well as the 2-D-NMR data of compounds **1**–**8** were compared to literature data and found to be in good agreement with reported values (Mat Ali et al., 1998; Chen et al., 2003; Wu et al., 2005; Yadav et al., 2012). Based on NMR data and molecular weight determined by HPLC-MS*,* compounds **9** and **10** were identified as two methyl ester derivatives of compounds **5** and **8**, respectively. ^13^C NMR data of compounds **9** and **10** are shown in Supplementary information together with the ^1^H NMR-spectra of compounds **1**–**4**. Structures of isolated compounds (**1**–**10**) are shown in Fig. 1.

### 3.3 Analysis of the NF-κB inhibitory effect of isolated compounds from *Oroxylum indicum*

**Fig. 4:**
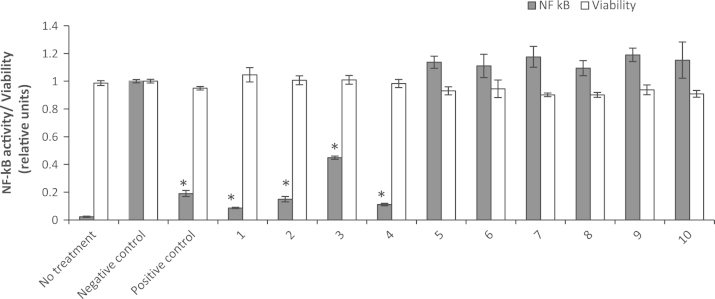
Effect of isolated compounds (30 µM) on NF-κB activity in HEK-293/NF-κB-luc cells; negative control: cells were treated which solvent vehicle (DMSO, 0.1%) and stimulated with TNF-α; positive control: cells were treated with parthenolide (5 µM) and stimulated with TNF-α. For compound assignment see Fig. 1. Data points represent mean±S.E.M.; *n*=3, ^⁎^*p*<0.05 (ANOVA/Tukey) compared with negative control.

Ten isolated compounds from the bark of *Oroxylum indicum* were analyzed in an NF-κB assay at a concentration of 30 µM. No decrease in cell viability within the used experimental setup could be observed. Interestingly, only the four flavonoid aglyca inhibited the NF-κB reporter gene transcription while the six flavonoid glycosides showed no significant activity (Fig. 4). The anti-inflammatory activity of compound **8**, baicalin, which is the glycoside of baicalein has been previously studied in several *in vivo* tests. Cytoprotective properties against injury to the liver caused by ischemia/reperfusion (Kim et al., 2010) and neuroprotective effects against ischemic reperfusion injury in rats (Xue et al., 2010) were reported to be related to inhibition of NF-κB. However, in an interleukin-1β (IL-1β) induced human retinal pigment epithelial cell line (Nakamura et al., 2003), baicalin neither inhibited IL-1β-induced IL-6 and IL-8 protein and mRNA production, nor did this flavonoid inhibit activation of NF-κB. Herein, we conducted the NF-κB assay with baicalein and baicalin simultaneously to demonstrate clearly the difference in activity between the flavonoid aglycone and its glycoside derivative *in vitro*. The result showed that baicalein was active while its glycoside, baicalin, did not suppress NF-κB activity in the HEK293/NF-κB-luc cell system. The same phenomenon was also observed with the glycosides of chrysin and oroxylin A and their corresponding aglyca. The increase in polarity and molecular weight due to the sugar moiety which negatively influences the penetration of cell membranes could be one explanation for the loss of activity of the glycosides.

### 3.4 IC_50_ determination of the active flavonoids

**Fig. 5:**
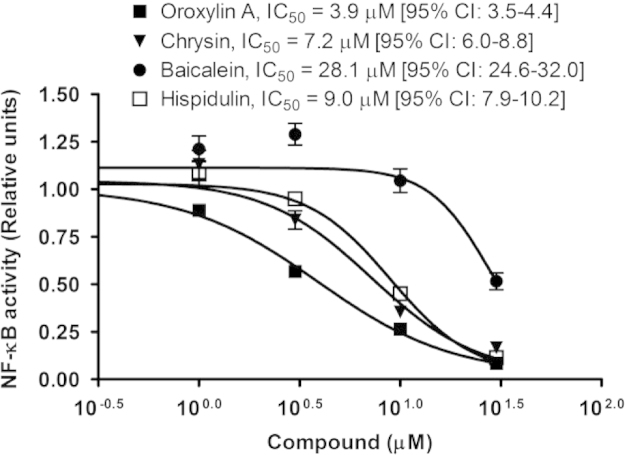
Dose–response curves and IC_50_ values of hispidulin, baicalein, chrysin and oroxylin A with respect to NF-κB activation.

In order to establish a preliminary structure-activity relationship and to assess the contribution of the four active flavonoids (hispidulin, baicalein, chrysin and oroxylin A) to the inhibitory effect on NF-κB activation of the *Oroxylum indicum* extract, IC_50_ values were determined. Oroxylin A was the most active substance (IC_50_=3.9 µM, 95% CI: 3.5–4.4 µM) while chrysin and hispidulin showed lower activity (IC_50_=7.2 µM, 95% CI: 6.0–8.8 µM, and 9.0 µM, 95% CI: 7.9–10.2 µM, respectively) in our cell based test system. Interestingly, baicalein revealed only a weak activity (IC_50_=28.1 µM, 95% CI: 24.6–32.0 µM) (Fig. 5). Baicalein, chrysin and oroxylin A are known for their anti-inflammatory effects attributed at least partially through the suppression of NF-κB activation (Chen et al., 2000; Kang et al., 2003; Ma et al., 2005; Shin et al., 2009; Sala et al., 2011) but this is the first time hispidulin was found to inhibit NF-κB activation. The results suggest that the presence of a hydroxyl group at the position 6 of the A ring (baicalein) reduces the activity of the flavone while a methoxyl group or no substitution at position 6 of the A ring (oroxylin A, hispidulin, chrysin) leads to a better inhibitory activity. Hispidulin possesses a similar structure to oroxylin A except for one additional hydroxyl group at position 4′ of the B ring. This modification results in a decrease of the ability to suppress NF-κB activation. These results supplement studies by Chen et al. (2004) who described the significance of hydroxyl groups at position 5 and 7 of the A ring and at position 4′ of the B ring for the effect of flavonoids in inhibiting ICAM-1, NF-κB and AP-1 activities on respiratory epithelial cells. Shin et al. (2011) studied the relationship among chalcone, flavanone, flavone and isoflavone derivatives and their inhibitory effect on TNF-α-induced NF-κB activation in HCT116 human colon cancer cell and emphasized the importance of hydroxyl substituents at C-5 position of the A ring of flavones.

### 3.5 Quantification of the active flavonoids (hispidulin, baicalein, chrysin and oroxylin A) in extracts and plant materials

Since baicalein was thought to be responsible for the anti-inflammatory activity of *Oroxylum indicum*, but showed only a weak NF-κB activity in this study, quantification of four flavonoids (hispidulin, baicalein, chrysin and oroxylin A) in the active extracts and stem bark materials was performed to get an idea about the contribution of each compound to the inhibitory effect on TNF-α-induced NF-κB activation. Validation of the established HPLC-DAD method is presented in Supplementary information. In the dichloromethane extract, hispidulin could not be determined; the contents of baicalein, chrysin and oroxylin A were 13.43%, 1.72%, 8.56% (w/w), respectively. These compounds were more abundant in the ethyl acetate extract: hispidulin (2.00%), baicalein (15.96%), chrysin (3.40%) and oroxylin A (13.85%, w/w). In both extracts the content of baicalein was higher than that of oroxylin A; however, the IC_50_ value of baicalein (IC_50_=28.1 µM) is almost 7 times higher than that of oroxylin A (IC_50_=3.9 µM). Therefore, it can be assumed that the NF-κB inhibitory activity of the dichloromethane and the ethyl acetate extracts is more driven by oroxylin A, although synergistic effects can never be ruled out. We also analyzed the content of the active flavonoids in four different *Oroxylum indicum* stem bark samples. The results (see Table 2) showed a strong variation in the different samples. In general, baicalein (0.13–0.45%) was the dominant flavonoid aglycone in the stem bark, followed by oroxylin A (0.09–0.14%).

*Oroxylum indicum* is used in many Asian countries; however, different parts of the plant are often used. The seeds of *Oroxylum indicum* are described in the Chinese Pharmacopeia (The State Pharmacopoeia Commission of P.R. China, 2010) while the stem bark is present in the Vietnamese Pharmacopoeia (The State Pharmacopoeia Commission of Vietnamese Pharmacopoeia, 2009). In the Ayurvedic system of medicine, the root bark is the officially used part of *Oroxylum indicum* (Radhika et al., 2011). Due to the popularity of this herbal medicine, several analytical procedures have been reported for the detection of flavonoids in different plant parts. Interestingly, hispidulin was not detected in the seeds (Krüger and Ganzera, 2012), while all four compounds (hispidulin, baicalein, chrysin and oroxylin A) were detected simultaneously in the root bark (Yadav et al., 2012). Among the quantified flavonoids, baicalein was the dominant flavonoid aglycone in all plant parts of *Oroxylum indicum*, 0.09–0.18% in the seeds (Krüger and Ganzera, 2012), 0.13–0.45% in the stem bark (Table 2), and 0.67% in the roots (Yadav et al., 2012).

## 4 Conclusion

Ethnopharmacological knowledge is a promising source for the selection of potential bioactive plant material. Among 17 Vietnamese plants used for the treatment of a variety of diseases associated with inflammation, the dichloromethane extracts of *Chromolaena odorata* and *Oroxylum indicum* proved to be promising inhibitors of the NF-κB pathway. A previous study identified fatty acids of *Chromolaena odorata* as inhibitors of NF-κB activation (Tran et al., 2011) but since the main constituents of this plant (Dat et al., 2009) are flavonoids, it might be necessary to consider a contribution of these compounds to the NF-κB inhibitory effect of this plant. Phytochemical studies of *Oroxylum indicum* bark led to the identification of four bioactive flavonoids (hispidulin, baicalein, chrysin, and oroxylin A). Baicalein and its glycoside, baicalin, are well known for their anti-inflammatory activity related to the inhibition of NF-κB activation. However, in this study, simultaneous testing with other constituents of *Oroxylum indicum*, baicalein showed a weak activity while baicalin was inactive, whereas other flavonoids *e.g.* oroxylin A seem to be more important in respect to the NF-κB inhibitory effect of *Oroxylum indicum* extracts. Altogether, the performed study supports the traditional use of some of the investigated species against inflammation by the found *in vitro* activity and identified the stem bark of *Oroxylum indicum* as a valuable source of flavonoids with an anti-inflammatory activity.

## Figures and Tables

**Table 1:** Latin names, plant parts and traditional uses of selected plant species in the screening for NF-κB inhibitors.

**Latin name**	**Family**	**Plant part**	**Voucher specimen**	**Traditional uses** (National Institute of Medicinal Materials, 2004)
*Abutilon indicum* (L.) Sweet	Malvaceae	Aerial part	DN101	Treatment of fever, rheumatism, dysuria, carbuncle
*Achyranthes aspera* L.	Amaranthaceae	Root, aerial part	DN102	Treatment of rheumatism, contusion, osteodynia, dysuria
*Barleria lupulina* Lindl.	Acanthaceae	Aerial part	DL101	Treatment of snake-bites, bleeding wounds, rheumatism
*Coptosapelta tomentosa* (Blume) Vahl.ex Heyne var. *dongnaiensis* (Pit.) Phamh.	Rubiaceae	Stem, root	KH081	Treatment of bleeding wounds, contusion or used as a tonic
*Dischidia rafflesiana* Wall.	Asclepiadaceae	Pitcher-shaped leaf	PQ101	Treatment of rheumatism, snake-bites, jaundice or used as a tonic
*Drynaria quercifolia* (L.) J.Sm.	Polipodiaceae	Rhizome	DN103	Treatment of rheumatism, osteodynia, dentagia
*Chromolaena odorata* (L.) syn.: *Eupatorium odoratum* L. King et Robinson	Asteraceae	Aerial part	LA103	Treatment of diarrhea, rheumatism, burns, skin wounds
*Ficus pumila* L.	Moraceae	Stem, leaf	BMT101	Treatment of rheumatism
*Gleditsia fera* (Lour.) Merr.	Caesalpiniaceae	Thorn		Treatment of carbuncle, osteodynia
*Ipomoea biloba* Forsk. syn.: *Ipomoea pes-carprae* (L.) Sweet	Convolvulaceae	Aerial part	PT101	Treatment of fever, rheumatism, edema, snake-bites
*Justicia gendarussa* L. syn.: *Gendarussa vulgaris* Nees	Acanthaceae	Aerial part	DL102	Treatment of osteodynia, rheumatism, jaundice, hives
*Leea rubra* Blume	Leeaceae	Root	BT101	Treatment of rheumatism
*Momordica cochinchinensis* (Lour.) Spreng.	Cucurbitaceae	Seed	DL103	Treatment of carbuncle, contusion, swelling
*Oroxylum indicum* (L.) Vent	Bignoniaceae	Stem bark	Oro-1, Oro-2, Oro-3, Oro-4	Treatment of allergy, inflammation, jaundice
*Parabarium micranthum* (Wall.) Pierre ex Spire	Apocynaceae	Stem	DN104	Treatment of rheumatism, osteodynia
*Scoparia dulcis* L.	Scrophulariaceae	Aerial part	DN105	Treatment of diabetes, hypertension, sore throat, used as an antidote
*Smilax glabra* Roxb.	Smilacaceae	Rhizome	DN106	Treatment of syphilis, acute and chronic nephritis, metal poisoning, rheumatism

**Table 2:** Amount of individual flavonoids in the active extracts (DCM and EtOAc extracts) and stem bark materials of different *Oroxylum indicum* samples (Oro-1 to Oro-4). Values shown in w% of air-dried plant material and relative standard deviations are in parenthesis (*n*=3).

	Hispidulin	Baicalein	Chrysin	Oroxylin A	Σ of flavonoids (%)
DCM extract	n.d	13.43%	1.72%	8.56%	23.71
EtOAc extract	2.0%	15.96%	3.42%	13.83%	35.21
Oro-1	n.d	0.13% (4.36)	0.04% (1.43)	0.14% (3.92)	0.31
Oro-2	0.01% (4.50)	0.26% (4.87)	0.05% (4.28)	0.19% (2.86)	0.51
Oro-3	0.02% (1.19)	0.22% (4.46)	0.07% (1.22)	0.10% (1.11)	0.41
Oro-4	0.03% (1.89)	0.45% (3.32)	0.15% (5.01)	0.09% (4.74)	0.71
