# Supplementary material for: Screening of Vietnamese medicinal plants for NF-κB signaling inhibitors: Assessing the activity of flavonoids from the stem bark of Oroxylum indicum
Source: J Ethnopharmacol. 2015 Jan 15;159:36–42. doi: 10.1016/j.jep.2014.10.012 (PMC4292993; doi:10.1016/j.jep.2014.10.012)
Supplement: Supplementary file 1 — Supplementary data [file mmc1.docx]

**Supplementary information**

**Screening of Vietnamese medicinal plants for NF-κB signalling inhibitors: Assessing the activity of flavonoids from the stem bark of *Oroxylum indicum***

Thi Van Anh Tran^a,c^, Clemens Malainer^b^, Stefan Schwaiger^a*^, Tran Hung^c^, Atanas G. Atanasov^b^, Elke H. Heiss^b^, Verena M. Dirsch^b^, Hermann Stuppner^a^

^a^ Institute of Pharmacy / Pharmacognosy, Center for Molecular Biosciences Innsbruck, University of Innsbruck, Innrain 80/82, 6020, Austria

^b^ Department of Pharmacognosy, University of Vienna, Althanstrasse 14, 1090 Vienna, Austria

^c^ Department of Pharmacognosy, Faculty of Pharmacy, University of Medicine and Pharmacy of Ho Chi Minh City, 41 DinhTienHoang street, Ho Chi Minh City, Vietnam

*Corresponding author. Tel.: +4351250758409; *E-mail address*: stefan.schwaiger@uibk.ac.at

**Table S-1**: Summary of validation data of the developed HPLC-assay using sample Oro – 1.

| Substance | Regression equation | Correlation coefficient | Range (µg/ml) | LOD (µg/ml) | LOQ  (µg/ml) | Precision  (intra-day) | Precision  (inter day) | Accuracy (high spike) | Accuracy (low spike) |
| --- | --- | --- | --- | --- | --- | --- | --- | --- | --- |
| Hispidulin | y = 18.689x + 11.209 | 0.9995 | 5 - 500 | 0.35 | 0.85 | * | * | - | - |
| Baicalein | y = 28.911x – 235.65 | 0.9997 | 5 - 500 | 1.05 | 3.25 | 2.68 % | 4.35 % | 103.9 % | 101.9 % |
| Chrysin | y = 29.202x -7.6736 | 0.9998 | 5 - 500 | 0.50 | 1.60 | 3.09 % | 1.44 % | 97.8 % | 103.5 % |
| Oroxylin A | y = 29.132x – 128.75 | 0.9996 | 5 – 500 | 0.45 | 1.50 | 3.13% | 3.92 % | 99.0 % | 100.4 % |

* hispidulin was not determined in sample Oro-1

LOD (limit of detection) and LOQ (limit of quantitation) were defined as 3-times and 10-times signal to noise ratio, respectively.

**Table S-2:** ^13^C-NMR chemical shifts of oroxylin A-7-*O*-β-D-glucuronide (**5**), oroxylin A-7-*O*-β-D-methyl glucuronide (**9**); baicalin (**8**) and baicalein-7-*O*-β-D-methyl-glucuronide (**10**) (δ in ppm; in MeOH-*d*_4_).

| Compound | **(5)** | **(9)** | **(8)** | **(10)** |
| --- | --- | --- | --- | --- |
| C-2 | 166.4 | 166.3 | 166.4 | 166.1 |
| C-3 | 106.0 | 106.0 | 105.6 | 105.6 |
| C-4 | 184.5 | 184.5 | 184.5 | 184.4 |
| C-5 | 154.3 | 154.0 | 148.1 | 148.2 |
| C-6 | 134.4 | 134.7 | 132.1 | 132.5 |
| C-7 | 157.9 | 157.7 | 152.7 | 152.7 |
| C-8 | 95.9 | 95.8 | 95.9 | 95.4 |
| C-9 | 154.4 | 154.4 | 151.4 | 151.4 |
| C-10 | 107.9 | 108.0 | 107.9 | 107.8 |
| C-1’ | 132.4 | 132.4 | 132.4 | 133.2 |
| C-2’ | 127.7 | 127.6 | 127.6 | 127.5 |
| C-3’ | 130.3 | 130.2 | 130.2 | 130.4 |
| C-4’ | 133.2 | 133.1 | 133.1 | 132.9 |
| C-5’ | 130.3 | 130.2 | 130.2 | 130.4 |
| C-6’ | 127.7 | 127.6 | 127.6 | 127.5 |
| O-Me | 61.4 | 61.7 |  |  |
| C- 1’’ | 101.0 | 101.7 | 102.5 | 102.0 |
| C-2’’ | 74.5 | 74.6 | 74.5 | 74.1 |
| C-3’’ | 77.4 | 77.4 | 76.7 | 76.5 |
| C-4’’ | 73.0 | 72.8 | 73.0 | 72.6 |
| C-5’’ | 76.7 | 76.8 | 76.9 | 76.4 |
| C-6’’ | 173.0 | 170.8 | 173.0 | 171.2 |
| CH_3_-O |  | 53.1 |  | 52.8 |

**Figure S1:** ^1^H-NMR-spetrum of Oroxylin A (measured in MeOH-*d*_4_; 300.13 MHz)

**Figure S2:** ^1^H-NMR-spetrum of Chrysin (measured in MeOH-*d*_4_; 300.13 MHz)

**Figure S3:** ^1^H-NMR-spetrum of Baicalein (measured in MeOH-*d*_4_; 300.13 MHz)

**Figure S4**: ^1^H-NMR-spetrum of Hispidulin (measured in MeOH-*d*_4_; 300.13 MHz)
